# Supplementary figures and images for: Music can be reconstructed from human auditory cortex activity using nonlinear decoding models
Source: PLoS Biol. 2023 Aug 15;21(8):e3002176. doi: 10.1371/journal.pbio.3002176 (PMC10427021; doi:10.1371/journal.pbio.3002176)

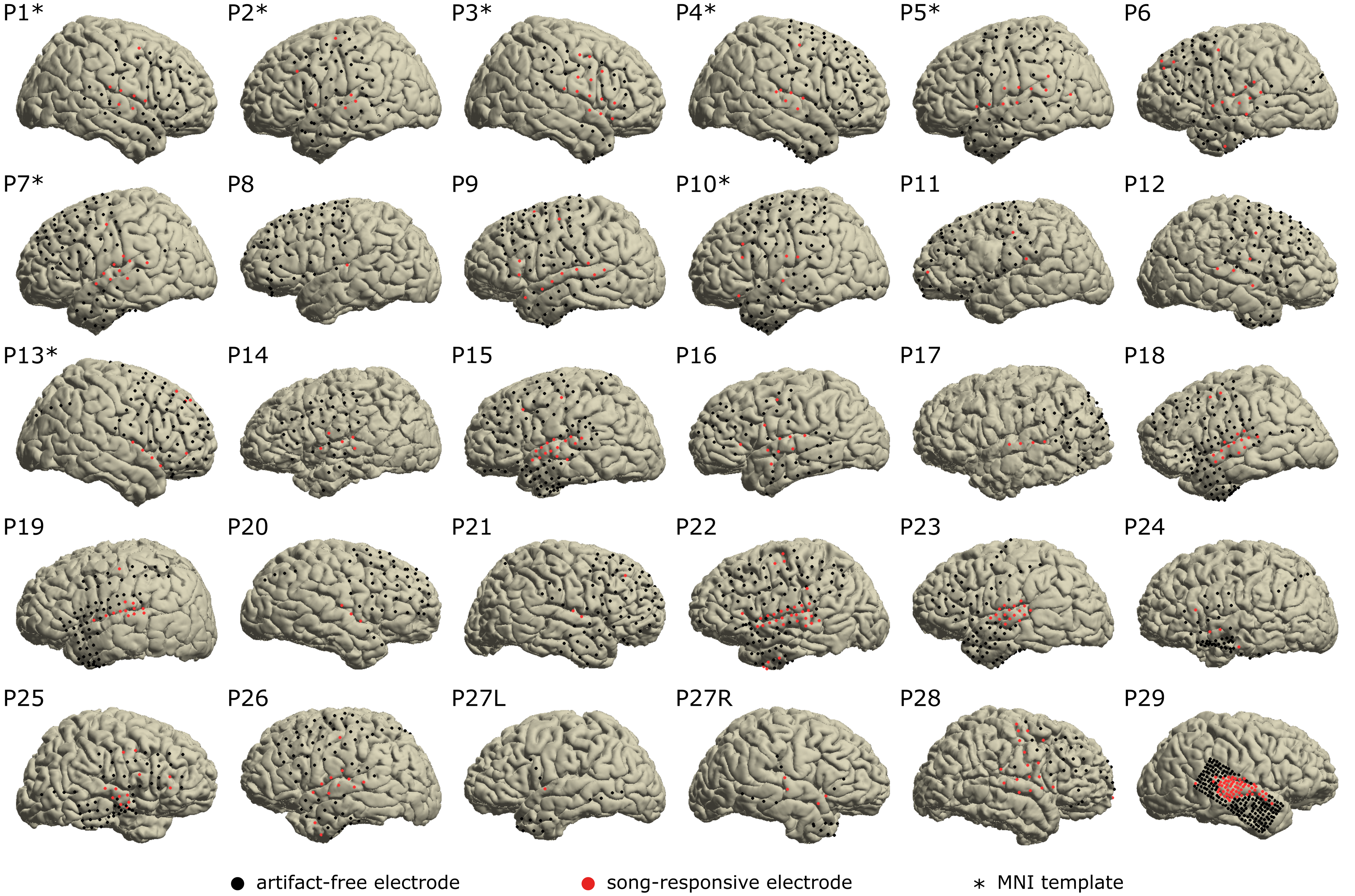

Supplement: S1 Fig — All presented electrodes are free of any artifactual or epileptic activity. Red marker color indicates song-responsive electrodes. For patients with low-resolution MR images, electrode coverage is plotted on the MNI template (asterisk symbols after patient code). Note that patient P27 had bilateral coverage. The data underlying this figure can be obtained at https://doi.org/10.5281/zenodo.7876019. (TIF) [file pbio.3002176.s001.tif]

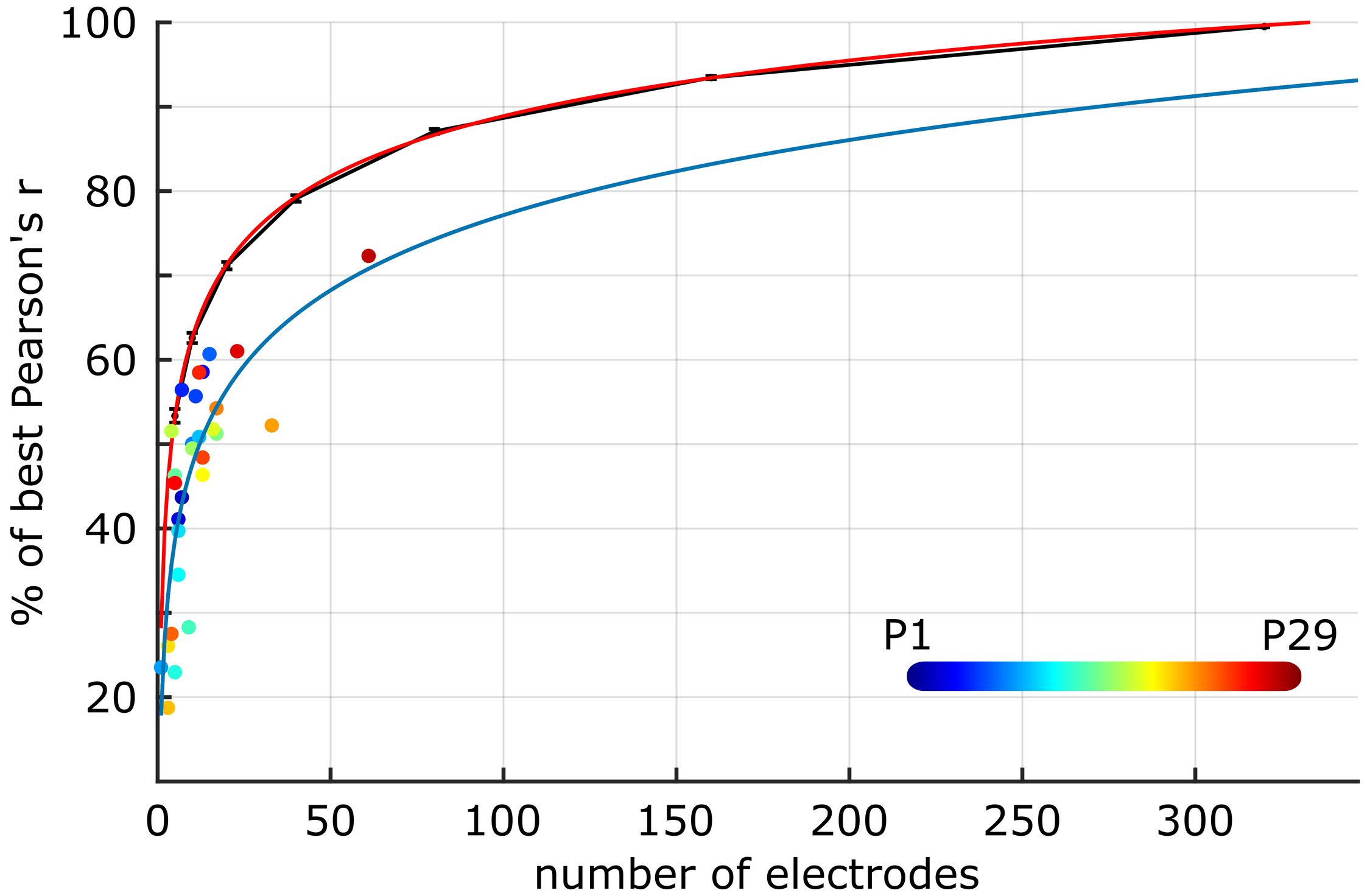

Supplement: S2 Fig — On the y-axis, 100% represents the maximum decoding accuracy, obtained using all 347 significant electrodes across all 29 patients. The black curve shows data points obtained from a 100-resample bootstrapping analysis, while the red curve shows a two-term power series fit line. Error bars indicate SEM. Colored dot markers represent prediction accuracy for single-patient decoding. For example, P1 had 7 significant electrodes used as features in the P1-only decoding models, which reached 43.7% of best prediction accuracy (dark blue dot). The blue curve shows a two-term power series line fitted on these single-patient prediction accuracy data points. The data underlying this figure can be obtained at https://doi.org/10.5281/zenodo.7876019. (TIF) [file pbio.3002176.s002.tif]

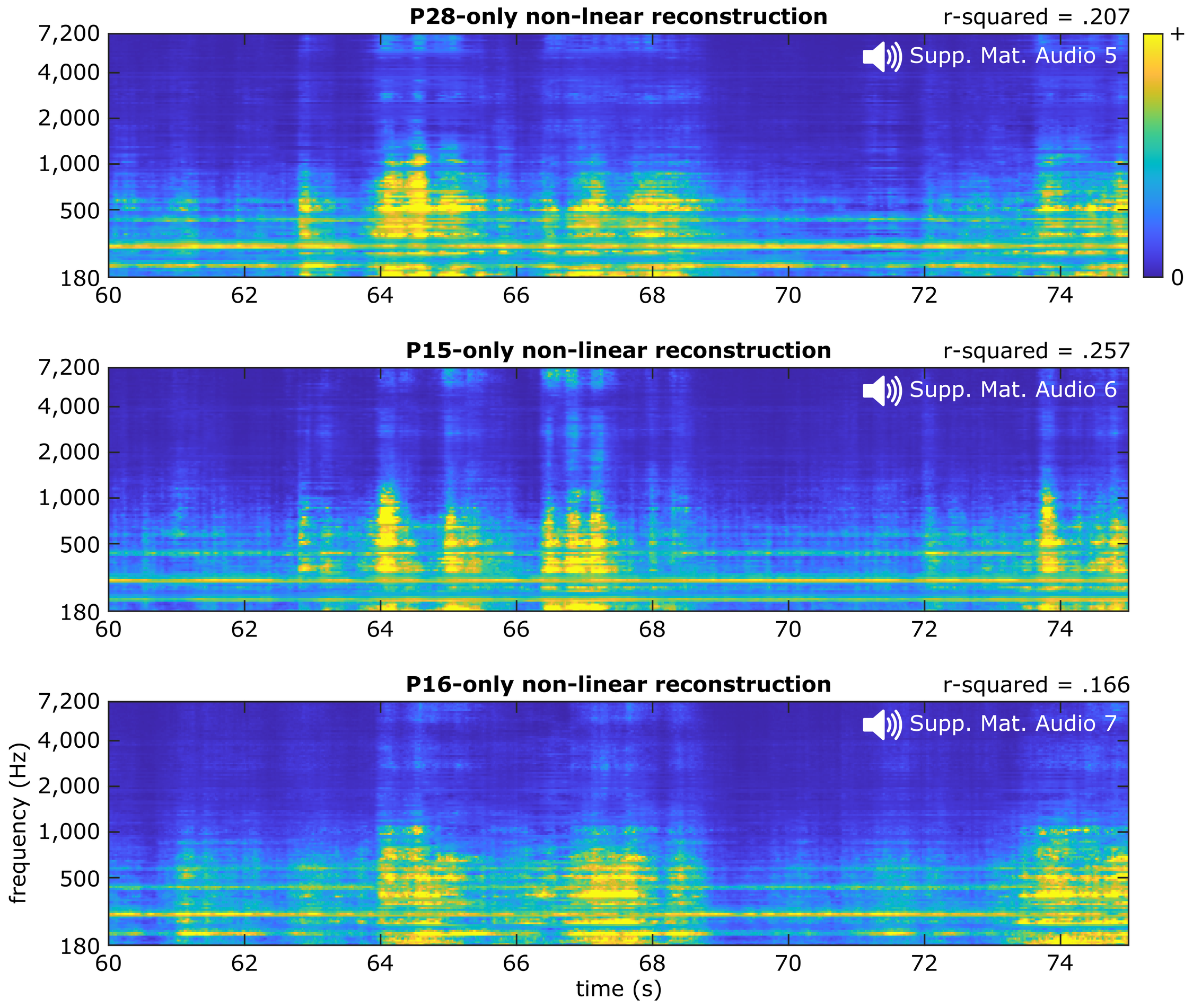

Supplement: S3 Fig — Auditory spectrograms of the reconstructed song using nonlinear models from electrodes of patient P28 only (top), P15 only (middle), and P16 only (bottom). Corresponding audio waveforms can be listened to in S5, S6, and S7 Audio files, respectively. The data underlying this figure can be obtained at https://doi.org/10.5281/zenodo.7876019. (TIF) [file pbio.3002176.s003.tif]

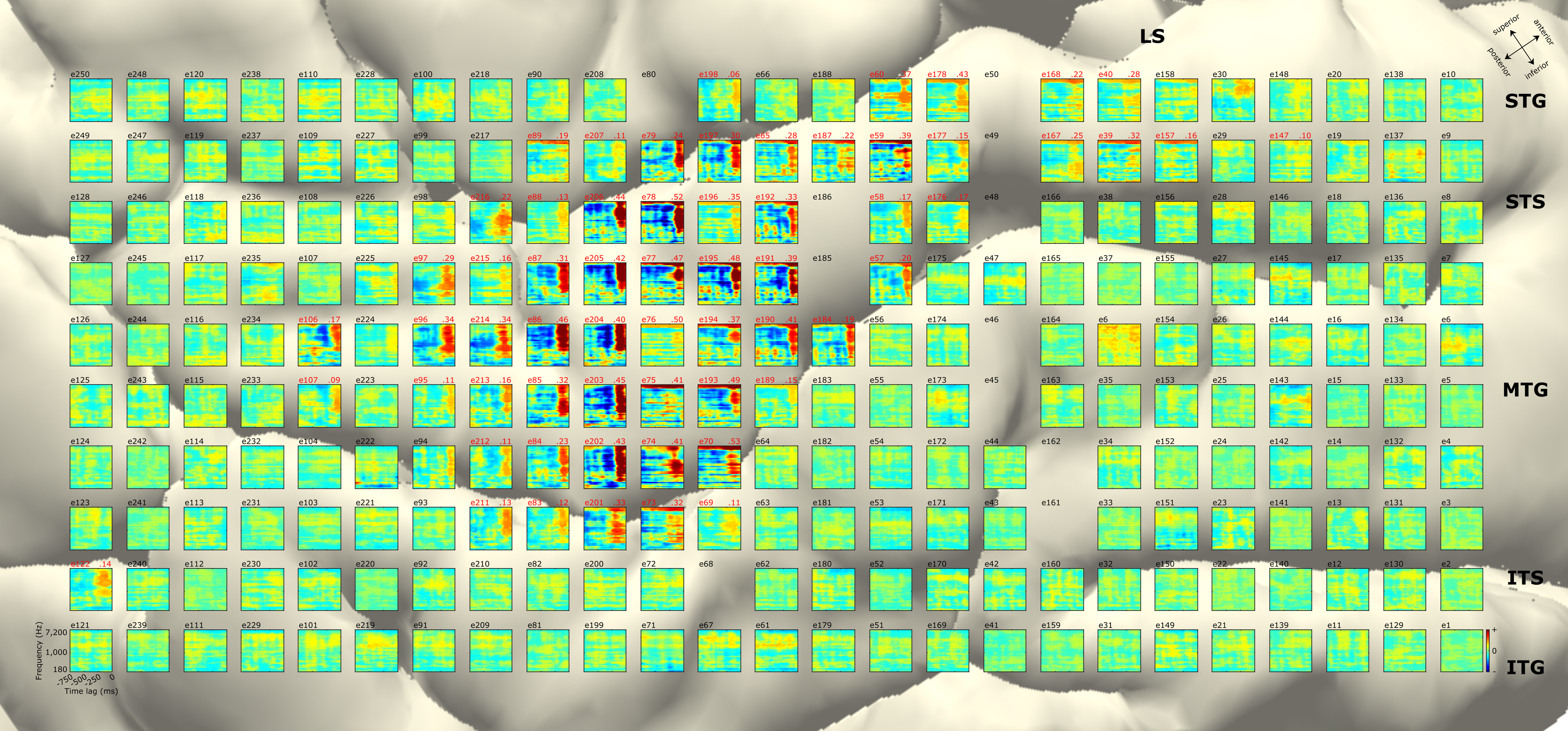

Supplement: S4 Fig — Red labels represent significant STRFs, and the respective prediction accuracies (Pearson’s r) are shown above each significant STRF on the right. Color code for STRF coefficients is identical to the one used in Fig 1. Anatomical axes are plotted in the top right corner (posterior on the left, in this right hemisphere coverage). Anatomical landmarks are shown as acronyms in bold font (ITS, inferior temporal sulcus; ITG, inferior temporal gyrus; LS, lateral sulcus, also called Sylvian fissure; MTG, middle temporal gyrus; STG, superior temporal gyrus; STS, superior temporal sulcus). The data underlying this figure can be obtained at https://doi.org/10.5281/zenodo.7876019. (TIF) [file pbio.3002176.s004.tif]
