# Supplementary material for: Music can be reconstructed from human auditory cortex activity using nonlinear decoding models
Source: PLoS Biol. 2023 Aug 15;21(8):e3002176. doi: 10.1371/journal.pbio.3002176 (PMC10427021; doi:10.1371/journal.pbio.3002176)
Supplement: Attachment — Submitted filename: responseToReviewers_LBellier.pdf [file pbio.3002176.s012.pdf]

Editor:

Based on the reviewers' feedback, we are happy to have you revise this work to address the reviewer concerns. When doing so, and given the broad scope of our journal, we'd also ask you to more explicitly discuss how these findings contribute to our broader understanding of human auditory processing mechanisms.

We thank the editor for this suggestion and have developed additional framing elements regarding a broader contribution of our results to the literature in the introduction (P3) and at the end of the discussion (P27).

P3, L46-54: “Despite extensive overlap with the speech perception network (17,18), some brain regions of the temporal and frontal lobes are preferentially activated during music perception (15,19–21). Recent studies report selective musical activation of different neural populations within the STG and the IFG (22). Both hemispheres are involved in music processing, with a relative preference for the right hemisphere compared to a left dominance for speech (23,24). However, an integrated view combining these musical elements and specific brain regions using a single predictive modeling approach applied to a naturalistic and complex auditory stimulus is lacking. In this study, we aimed to specify which brain regions are preferentially engaged in the perception of different acoustic elements composing a song.”

P27, L498-502: “The anatomo-functional organization reported in this study may have clinical implications for patients with auditory processing disorders. For instance, the musical perception findings could contribute to development of a general auditory decoder that includes the prosodic elements of speech based on relatively few, well-located electrodes.”

Besides, we added a new reference (P6, L109-110) and updated the reference gender statistics (P43, L828-831).

Reviewer #1: Bellier and colleagues analyzed a previously collected iEEG dataset in 29 pharmacoresistant epileptic patients who were implanted with ECoG electrodes prior to neurosurgery, with high-frequency neural activity measured while passively listening to a popular Pink Floyd's song. Using combined regression-based decoding models and encoding analyses, they successfully reconstructed multidimensional, acoustic song features from recorded neural activity. These results are both original and important, as they reveal a) information processing content in the STG during music perception, showing the feasibility of reconstructing perceived music from high-frequency neural activity, along with b) a new STG sub-region tuned to musical rhythm encoding.

While previous published studies have applied decoding models to decode music-induced neural activity with a classification approach, the present study is the first to show the reconstruction of music through regression-based decoding models, by analogy to previous work with speech stimuli (AnumanchipalliG et al. Nature 2019).

The manuscript is clearly written and the methodology and research questions are sound. However, several issues require further clarification.

Essential points:

1. Fig. Supp. 1 shows that some patients had vastly more song-responsive electrodes than others (e.g., P22, P23, P29). In fact, in Fig. Supp. 2 it is quite clear that some patients have rather low decoding accuracy. How does the number of song-responsive electrodes affect decoding accuracy in each patient? Would removing data of any patients with a low number of song-responsive electrodes and/or low decoding accuracy leave the overall decoding results unaffected?

We thank the reviewer for this excellent question. As shown by the ablation analysis, it is not the number of song-responsive electrodes that matters but rather their location and encoding model score. We reconstructed the song from HFA for three additional patients (new Fig. Supp. 3), with 23, 17 and 10 song-responsive electrodes, and a reconstruction accuracy of .21, .26 and .17, respectively, which illustrates this point (P14, see next response).

The authors make a case on the feasibility of predictive modeling even in individual patients, but since they show it only in the patient with the most song-responsive electrodes in the right hemisphere (P29), it is not clear to what extent the results of the present study are representative of only a subset of patients or the group in general.

We thank the reviewer for the opportunity to address the single-patient applicability. We followed their suggestion and reconstructed the song from neural activity of 3 more single patients (patients P28, P15 and P16). We chose these patients because they had fewer electrodes than P29 (23, 17 and 10, resp., as opposed to 61 in P29), with a lower electrode density (1 cm, 6 mm and 1 cm center-to-center, resp., as opposed to 3 mm in P29), but with good song-responsive regional coverage (mostly right, left and left STG, respectively, with few electrodes in SMC and IFG) and still with a good linear decoding accuracy (Pearson's r=.387, .322 and .305, resp. as opposed to .45 in P29; 2nd, 12th and 15th best performers, resp.). Non-linear decoding models reconstructed the song spectrogram with an effective r-squared of .207, .257 and .166, respectively. Interestingly, we retrieved the vocals and harmony in the reconstructed waveforms, with variable recognizability. Altogether, these findings strengthen our claim that predictive modeling is feasible at the individual patient level even with low-density grids and a few well-located electrodes. We added these new single-patient reconstruction results in the manuscript (P14, Fig. Supp. 3 on P50—relegating former Fig. Supp. 3 to Fig. Supp. 4, P16; and Supp. Mat. Audio 6, 7 and 8).

<u>P14, L252-263:</u> "To assess the lower bound of single-patient-based decoding, we reconstructed the song from the neural activity of three additional patients (P28, P15 and P16), with fewer electrodes (23, 17 and 10, respectively, as opposed to 61 in P29) and a lower density (1 cm, 6 mm and 1 cm center-to-center electrode distance, respectively, as opposed to 3 mm in P29), but still covering song-responsive regions (mostly right, left and left STG, respectively) and with a good linear decoding accuracy (Pearson's r=.387, .322 and .305, respectively, as opposed to .45 in P29). Non-linear models reconstructed the song spectrogram with an effective r-squared of .207, .257 and .166, respectively (Fig. Supp. 3). In the reconstructed waveforms (Supp. Mat. Audio 6, 7 and 8), we retrieved partial vocals (e.g., in P15, "all", "was", "just a brick" were the only recognizable syllables, as can be seen in the reconstructed spectrogram, Fig. Supp. 3, top) and a sense of harmony, although with varying focus in recognizability."

P16, L286-287: "[…] see Fig. Supp. 4 for a detailed view of patient P29's STRFs computed for their 10-by-25, 3mm-center-to-center grid of electrodes)."

2. Methods, pp.29-31: encoding analysis. The authors made a number of decisions on parameter values in order to prevent model overfitting. While this reflects good practices, it does not guarantee that the problem of overfitting will be eliminated entirely. Can the authors provide an estimate on the degree of protection against overfitting in their model? Have they tested it against a range of different parameter values?

We appreciate and share the reviewer's concern regarding overfitting, as without proper measures this could impact the validity of our present claims. We implemented overfitting protection at different steps of our modeling pipeline. These include: 1. assessing autocorrelation of the neural and audio time series to enable group splitting into training, validation and test sets, 2. fitting the scaler on the training set and applying it to the validation and test sets, 3. using early stopping for linear models and L2 regularization for non-linear models, and 4. ensuring all models were evaluated on held-out test sets. Regarding group size, the figure below shows the autocorrelation in the stimulus spectrogram (left) and in the HFA (right). In both signals, autocorrelation rapidly decreases within the first 50 ms, and reaches a plateau at about 250 ms. With the sampling rate of 100 Hz, it suggests that allowing examples (i.e., a set of time-lagged features and the related target value) up to 5 samples apart to be split into distinct sets (e.g., one in the training set and the other in the validation set) is equivalent to testing the model on the training data, and would render early stopping inefficient at preventing overfitting.

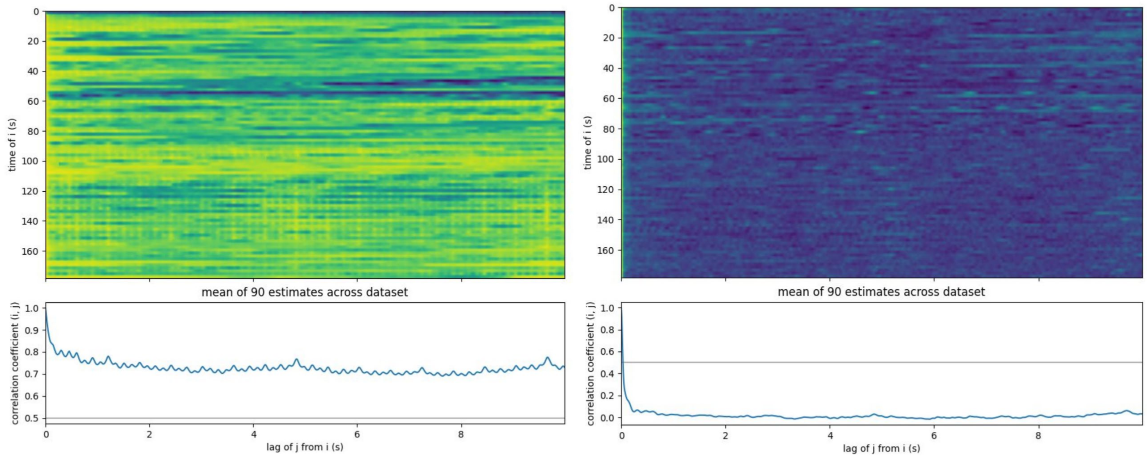

Regarding early stopping, the figure below shows our optional model fitting graphical output for early stopping, where in the top-left panel we see, for one resample, that the linear model stopped training once the validation error started increasing, while the training error was still decreasing at a steady rate. This effectively prevents overfitting.

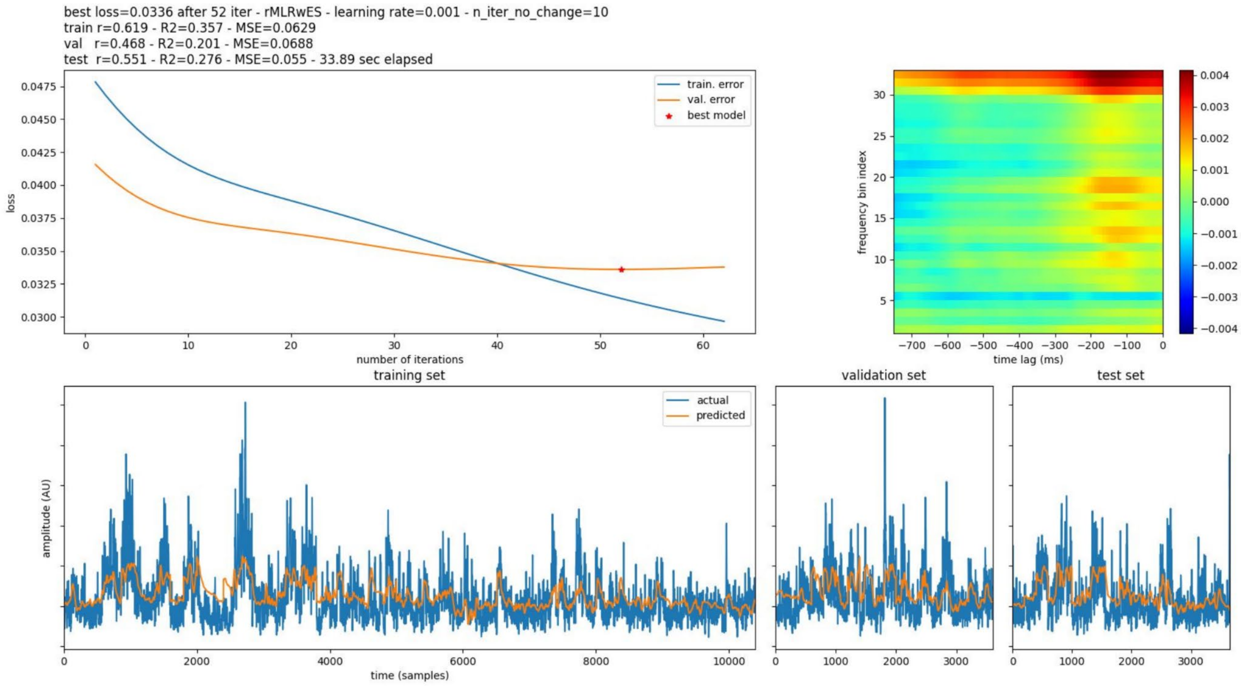

To clarify these points in the manuscript, we added the following paragraph at the end of the methods section (P42). Our models yield biologically plausible results supporting that our measures for preventing overfitting were successful. For example, in Fig. Supp. 4, all significant electrodes are on STG, significant STRFs have a shared coefficient structure as illustrated by the ICA components in Fig. 5 and all other non-significant STRFs lack any structure.

P42, L809-821: "**Preventing overfitting.** Given the claims of this paper are based on the results of encoding and decoding models, it was crucial to make sure we avoid overfitting. We implemented state-of-the-art practices at all steps. Before splitting, we assessed autocorrelation in both the stimulus and the neural data time series and defined 2-second groups of consecutive samples as indivisible blocks of data to be allocated to either the training, validation or test set (5-second groups for song reconstruction). For data scaling, we fitted the scaler on the training set, and applied it to both the validation and test sets (scaling the whole time series, as sometimes seen in the literature, allows the scaler to learn the statistics of the test set, possibly leading to overfitting). Most importantly, we used early stopping for our encoding and decoding models, which by definition stops training as soon as the model starts to overfit and stops generalizing to the validation set; and L2 regularization for our non-linear models, which constricts model coefficients to prevent overfitting. Finally, we made sure to evaluate all models on held-out test sets."

3. Results, p.20: "Furthermore, the fact that removing single regions in the left hemisphere had no impact but removing all left electrodes did, suggests redundancy within the left hemisphere, with musical information being spatially distributed across left hemisphere regions."

Could it be in turn that there is a mass effect, such that removing all left electrodes leads to a sharp drop in decoding accuracy due to insufficient remaining patient/electrode data? This may be the case because there were overall more left than right unilateral patients and overall more song-responsive electrodes in the left hemisphere than in the right hemisphere.

We thank the reviewer for this alternative explanation. After careful consideration we do not think this is likely for the following reasons. First, we found that removing 167 electrodes (with a sustained component) for the full set of 347 song-responsive electrodes did not impact decoding accuracy. As such, removing 133 left electrodes should not necessarily be accompanied by a loss of accuracy. Second, the decoding model cares about information represented in the predictors rather than in the number of predictors. This is illustrated by the individual patient decoding findings, showing that 61 electrodes suffice to provide the information leading to reconstructing a recognizable song with high prediction accuracy, not far from the reconstructed song from all 347 song-responsive electrodes. Our findings also show that removing all left electrodes is followed by a decrease in accuracy because the left hemisphere represents musical information in a distributed and redundant way.

On a related question, with respect to Fig.3A, it would be important to know if the anatomical location of the electrodes that provide best prediction accuracy matters (for example, where are the 43 electrodes that contribute to the 80% best prediction accuracy concentrated in the bootstrap analysis?).

In this analysis, the selection of the electrodes included in the model were bootstrapped across patients. Bootstrapping was done by randomly drawing sets of n electrodes 100 times for each n, with n in 5, 10, …, 320. Note that this bootstrap analysis is blind to anatomical location in the way it draws sets of electrodes. This means that the 43 electrodes referred to by the reviewer are not a set of specific electrodes, but rather the number of electrodes determined to reach 80% best prediction accuracy. We agree with the reviewer and note in the paper that for decoding accuracy, localization (and quality) matters more than quantity, based on the results of the ablation analysis. We explicitly note that the anatomical location was disregarded during the bootstrap analysis in Fig. 3A (P12 and P35).

P12, L219: "We performed a bootstrap analysis by fitting linear decoding models on subsets of electrodes randomly sampled from all 347 significant electrodes across the 29 patients, regardless of anatomical location."

P35, L658-660: "In a bootstrapping approach, we randomly constituted subsets of 5, 10, 20, 40, 80, 160 and 320 electrodes (sampling without replacement), regardless of their anatomical location, to be used as inputs of linear decoding models."

Other points:

4. Introduction, p.3: The intricate issue of overlap of brain structures and hierarchical information structure between music and speech is dismissed somewhat superficially, with reference only to a couple of relatively older citations. A more up-to-date and balanced view should be provided.

We thank the reviewer for this good point. We have now extended our introduction on the structures involved in music and speech processing (P3) and expanded on the comparison of our results to the speech literature (P24).

P3, L46-54: "Despite extensive overlap with the speech perception network (17,18), some brain regions of the temporal and frontal lobes are preferentially activated during music perception (15,19–21). Recent studies report selective musical activation of different neural populations within the STG and the IFG (22).

Both hemispheres are involved in music processing, with a relative preference for the right hemisphere compared to a left dominance for speech (23,24). However, an integrated view combining these musical elements and specific brain regions using a single predictive modeling approach applied to a naturalistic and complex auditory stimulus is lacking. In this study, we aimed to specify which brain regions are preferentially engaged in the perception of different acoustic elements composing a song."

P25, L426-429: "Note that the overall correlation coefficient computed across all 29 participants for linear decoding models in our present study (r=.29, from the non-normalized values of Fig. Supp. 2) is comparable to the overall decoding accuracy of linear models for pure speech stimuli (r=.28, across 15 participants) (34), suggesting that this limitation is shared between speech and music."

5. Methods: p.26: "Eight patients had more than one recording of the present task, in which cases we selected the cleanest one (i.e., containing the least epileptic activity or noisy electrodes)."

Information on frequency and distribution of epileptic activity in each patient's data should be provided, along with proportions of data deletion.

We agree with the reviewer that a detailed, patient-wise quantification of electrodes exhibiting epileptic activity (or even of noisy electrodes) was missing in the manuscript. We added this information on P30, and thank the reviewer for suggesting this.

P30, L557-560: "Overall, from our starting set of 2,668 electrodes, we removed 106 noisy electrodes (absolute range 0-22, mean 3.7 electrodes; relative range 0-20.2%, mean 3.7%) and 183 epileptic electrodes (absolute range 0-28, mean 6.3; relative range 0%-27.6%, mean 7.6%), and obtained a set of 2,379 artifact-free electrodes."

6. Results, p.7: the higher proportion of song-responsive electrodes in the right hemisphere is presented as significant in a chi-square test. However, from inspection of Fig.2A it appears that this result could be biased by the higher concentration of left-hemispheric electrodes in regions that are minimally responsive to song stimuli, such as the left temporal pole and left DLPFC. What if this analysis were restricted to regions of interest, like auditory cortices? Despite the limited contribution of this finding to the overall picture, some rephrasing is needed with respect to this point.

We concur that different non-uniform coverages in left and right hemispheres could bias this chi-square test. Following the reviewer's suggestion, we restricted this analysis to the three main song-responsive regions (STG, SMC and IFG), and observed a stronger effect (P9). We thank the reviewer for highlighting this potential bias.

P9, L171-178: "To examine whether the higher proportion of song-responsive electrodes in the right hemisphere was driven by different non-uniform coverages between both hemispheres (e.g., by a denser coverage of non-auditory regions in the left hemisphere than in the right hemisphere), we restricted our analysis to the three main song-responsive regions (STG, SMC and IFG). We found a higher proportion of song-responsive electrodes in these right song-responsive regions, with 133 significant electrodes out of 374 total, against 165 out of 654 in the corresponding left regions (35.6% versus 25.3%, respectively; X^2 (1, N=1,026) = 12.34, $p < .001$)."

7. Decoding Ablation analysis: why did the authors use a linear rather than a non-linear decoding model for the ablation analysis?

This is a great question, and we thank the reviewer for drawing our attention to an aspect we did not adequately explain in the manuscript. The main reason we used linear decoding models in the ablation analysis is for interpretability. Deep neural networks can model any function and can compensate for ablations more than linear models, potentially masking effects of interest. For example, assuming SMC represents a nonlinear transformation of the acoustic information represented in STG, and we ablate STG, then a nonlinear model could reconstitute the acoustic information from the higher-order, nonlinear information in SMC and prevent us from seeing any impact from this ablation on decoding accuracy. Using linear decoding models restricts compensation to the same acoustic level allowing to drawing conclusions from the ablation analysis. Nonlinear models are more computationally intensive to fit and require tuning of more hyperparameters, the values of which could differ between ablations, potentially biasing the results. We added this statement in the manuscript (P41).

P41-42, L799-807: "Note that we performed this ablation analysis on linear decoding models to ensure interpretability of the results. Indeed, as deep neural networks are able to model any function (38), they could reconstitute acoustic information (e.g., when ablating STG) from higher-order, non-linear representation of musical information (e.g., in SMC or IFG), and could thus alleviate, if not mask entirely, any impact on decoding accuracy. Using linear decoding models restricts compensation to the same (or at most, linearly related) information level, and enables drawing conclusions from the ablation analysis results. Further, compared to linear models, nonlinear models require tuning more hyperparameters, with most likely different optimal values between ablations, which could bias the results."

Reviewer #2: I'm glad the authors pursued this study. I have been wondering about this.

This manuscript is a good contribution to the literature.

I have a couple of minor nits.

1. It might be worth stating up front that this paper is reconstructing a full spectrogram, not the envelope (and other features) that are usually done for (EEG) speech decoding.

We thank the reviewer for suggesting this clarification, and edited the manuscript at several places to explicitly state that we are reconstructing the auditory spectrogram (P5, P7).

P5, L94-95: "We investigated to what extent the auditory spectrogram of the song stimulus could be reconstructed from the elicited HFA using a regression approach."

P7, L133-135: "In summary, we used regression-based decoding models to reconstruct the auditory spectrogram of a classic rock song from the neural activity recorded from 2,668 ECoG electrodes implanted in 29 neurosurgical patients […]."

2. Why isn't ridge regression directly used for the decoding? This seems more reliable than a method based on SGD.

For both linear encoding and linear decoding models, we used RMSProp with early-stopping providing robust prediction accuracy in the fraction of the time needed by ridge regression (minutes instead of hours for computing the 250 resamples of training, validation and test sets). RMSProp was easier to implement at scale without any hyperparameter to tune (as opposed to the L2 norm regularization parameter, alpha, in ridge regression, which then calls for an inner cross-validation loop). Early-stopping was also used in previous studies in the literature applying a similar predictive modeling approach (Pasley et al., 2012; Martin et al., 2014). Note that during our initial attempts with ridge regression, we found similar results than the ones obtained using our RMSProp with early-stopping implementation, in that both models yielded similar STRFs and prediction accuracies all while successfully preventing overfitting.

3. For comparison purposes it would be good to mention the decoding accuracy (either envelope or spectrograms) for pure speech data. Do the authors have that from previous studies?

In a similar speech analysis study (Pasley et al., 2012), 15 participants passively listened to pure speech (isolated words or sentences) and by reconstructing the auditory spectrogram using linear models, the authors found an overall correlation coefficient of r=.28. In our present study, the overall decoding accuracy of linear models across all 29 participants is r=.29 (computed from the non-normalized values of the individual Pearson's r in Fig. Supp. 2). We added a mention to this comparison in the discussion (P24) and thank the reviewer for this great suggestion.

P24, L426-429: "Note that the overall correlation coefficient computed across all 29 participants for linear decoding models in our present study (r=.29, from the non-normalized values of Fig. Supp. 2) is comparable to the overall decoding accuracy of linear models for pure speech stimuli (r=.28, across 15 participants) (34), suggesting that this limitation is shared between speech and music."

It is nice to see the ICA analysis of STRFs, and the resulting distributions of types.

Good job.

Reviewer #3: Bellier et al. carry out an interesting study centered around the reconstruction of music stimuli from human electrophysiology data. The manuscript is well written, the analyses are rigorous and well described. I only have a few of minor remarks.

L[ine] number: [original text] comment/proposed edit

L27: [recognizable song] the term "recognition" is used throughout the manuscript to indicate that song segments reconstructed from brain activity were more strongly correlated (in the spectral domain) with

the audio signal of the same song segment than with audio signals from different song segments. This correlation approach is legitimate, and indeed addresses in some form the confusability of the reconstructions with different segments of the same song. However, I wonder whether "recognizable song" might be completely appropriate, as it implies an experiment assessing recognition with human listeners, experiment that was not carried out by the authors.

We clarified our definition in the introduction (P5). In the correlation approach described by the reviewer, we quantified recognizability and referred to the results as song-excerpt identification. Regarding experiments assessing involving human listeners, the adjective "intelligible" has been used in the literature (Pasley et al., 2012; Akbari et al., 2019), which is why we avoided it and used "recognizable" instead.

P5, L102-104: "[…] wherein only nonlinear models provide a recognizable reconstructed stimulus (i.e., a song that a listener could identify, without the vocals being necessarily intelligible) […]"

L47: [approaches] approach

We thank the reviewer for catching this and we made the edit (P3, L52).

L88: [While investigating to what extent the song stimulus could be reconstructed from the elicited HFA using a regression approach] why did you focus on HFA only? This needs to be justified, as low-frequency brain signals can indeed contain a lot of information about time-varying acoustic structure.

This is a good point, and we added a sentence in the discussion to address this limitation (P27). With encoding models, linear and nonlinear decoding models, and different higher-order analyses building upon these models, we restricted the scope of this present study to HFA on the neural activity side, and an auditory spectrogram of the song on the stimulus side. We fully agree with the reviewer that other estimates of neural activity are worth studying, which we are pursuing in a follow-up study.

P27, L503-509: "We limited our investigation to the auditory spectrogram on the stimulus side, and to HFA on the neural activity side given the complex study design encompassing several higher-order analyses building upon encoding and decoding models. Future studies should explore different higher-order representations of musical information in the auditory brain (i.e., notes, chords, sheet music), as well as lower neural oscillatory bands and spectral components (e.g., theta, alpha and beta power, aperiodic component), known to represent relevant acoustic information, adding another brick in the wall of our understanding of music processing in the human brain."

L89: [three factors on reconstruction accuracy: (1) model type (linear versus nonlinear), (2) electrode density (the number of electrodes used as inputs in decoding models), and (3) dataset duration.] This appears more like a manipulation conceived to assess the performance of the reconstruction method rather than a manipulation designed to answer specific questions about the function of the auditory system.

We understand the reviewer's impression upon reading this sentence, and added a clarification in the text (P5). While these manipulations provide methodological insights, (1) and (2) also tell us about the auditory brain (respectively, about availability of acoustic information in STG and upstream; and about the concentration of neural populations representing musical information in a few specific subregions).

P5, L95-98: "We also quantified the effect of three factors on reconstruction accuracy: 1. model type (linear versus nonlinear), 2. electrode density (the number of electrodes used as inputs in decoding models), and 3. dataset duration, to provide both methodological and fundamental insights into the reconstruction process."

L112: [Here we investigated whether we could observe similar HFA activity profiles, namely onset and sustained, in response to a musical stimulus.] Why is this particular analysis important to our knowledge of the auditory system?

An important effort in the field of auditory neuroscience aims at determining to what extent speech and music processing overlap in the brain. Assessing whether music perception is supported by similar HFA activity profiles than speech is important. We added more motivation for this analysis in the text (P6).

P6-7, L127-131: "We hypothesized that the right STG would have a primary role in representing acoustic information during music perception, and that we would observe a similar antero-posterior STG parcellation with sustained and onset responses as observed in the speech domain. Further, we anticipated that other components, tuned to specific musical elements, might emerge and extend this parcellation."

L118: [and that we would observe some degree of STG parcellation with similar structure as observed in the speech domain.] Can you be more specific? "Some degree of STG parcellation" is perhaps too undefined.

Thank you for underlining this lack of clarity. We have now reworded this section (P6) to make our statement more specific.

P6-7, L129-130: "[…] that we would observe a similar antero-posterior STG parcellation with sustained and onset responses […]"

L162: [Analysis of STRF prediction accuracies (Pearson's r) found a main effect of laterality (two-way ANOVA] Specify whether the two-way ANOVA considers simultaneously the effects of cortical region and hemisphere. Was the interaction term modelled? If not, why?

We used an additive two-way ANOVA with laterality and cortical regions as factors. We predicted a right > left laterality effect, classic in the literature of music processing, and that the STG would be more linearly related to the song acoustics than SMC or IFG, regardless of laterality. We did not hypothesize any particular interaction between these two independent variables (nor did we have a strong hypothesis that there would be no interaction), and we did not include the interaction. We thank the reviewer for leading us to improve clarity on this point and added this specification on P9.

P9, L180-183: "Analysis of STRF prediction accuracies (Pearson's r) found a main effect of laterality (additive two-way ANOVA with laterality and cortical regions as factors; $F(1, 346) = 7.48$, $p = 0.0065$; Fig. 2D), with higher correlation coefficients in the right hemisphere than in the left (MR = .203, SDR = .012; ML = .17, SDL = .01)."

L175: [over 250 resamples] resamples of what?

We resampled the training, validation and test sets, leading to 250 distinct models for each electrode. We added more details in the figure legend (P10).

P10, L193-194: "Significance was determined by the STRF prediction accuracy bootstrapped over 250 resamples of the training, validation and test sets."

L185: [100-resample bootstrapping analysis] resampling of what?

Here, we resampled 100 times sets of electrodes (without replacement) for each number of electrodes. We added more details in the figure legend (P11) so that the reader will not have to go to the methods to understand this point.

P11, L204-205: "The black curve shows data points obtained from a bootstrapping analysis with 100 resamples for each number of electrodes (without replacement), while the red curve shows a two-term power series fit line."

L266: [explaining more than 5% variance] EACH explaining more than 5% variance. Also, if I read the labels on panel B well, it might be better to indicate that the 3 ICs explained, together, more than 50% of the variance of the significant STRFs (same comment for line 260).

We thank the reviewer for this suggestion and added this to the manuscript (P16 and P39).

P16, L289-291: "We identified three components with distinct spectrotemporal tuning patterns, each explaining more than 5% variance, and together explaining 52.5% variance (Fig. 5B)."

P17, L295-296: "Three ICA components each explaining more than 5% variance of all 347 significant STRFs."

P39, L753-754: "We found 3 ICA components each explaining more than 5% of variance, and together explaining 52.5% variance."

Fig. 5, panels C and E: the coefficient maps are hard to parse. Would it be possible to show the positive coefficient sensors on top of the negative (black) coefficients? Alternatively, you could show only the positive coefficient sensors here, and put in supplementary materials a figure where you show all sensors.

We welcome the reviewer's perception of Fig. 5C and E, and improved readability of the coefficient maps by reducing the marker size of electrodes with a negative coefficient (in black), by slightly shifting these

electrodes medially to have them plotted below electrodes with positive coefficients, and by increasing the marker size of electrodes with a positive coefficient (P17).

P17, L297-299: "Color code indicates coefficient amplitude, with in red the electrodes which STRFs represent the components the most."

Fig. 6, panel C: the correspondence between the time-varying correlations and the musical structure (vocals, lead guitar, synth) is hard to parse, as one needs to project mentally the coloured horizontal bars in panel A onto each of the time-varying correlation plots. Would it be possible to replicate the coloured bars in panel A on top of each of the three correlation plots?

We thank the reviewer for this suggestion and updated Fig. 6 to improve readability (P19).

L311, L323: [referred from] referred to from

We corrected this grammatical error in both lines and thank the reviewer for catching this (P20, L342 and 354).

L384: [music perception] music representation or processing would perhaps be more appropriate. Same for other instances.

In our manuscript, we use the term music perception as a subset of music processing, which encompasses the processing of musical information at both the perception and production stages, and music representation as the neural code supporting music processing. Note that this view of perception versus production is also present in the literature (e.g., Koelsch & Siebel, 2005; Zatorre & Salimpoor, 2013). However, we also agree that "music perception" can be interpreted in a more granular, psychological, phenomenological way, as the construction of a musical percept from neural activity, or in other words, from the neural representation of music to a musical percept (while in our definition it would be from a musical sound all the way to a musical percept). Therefore, we added our broader definition immediately after the first instance of "music perception" in the introduction (P2), and thank the reviewer for helping us improve clarity.

P2, L38-41: "Understanding the neural substrate supporting music perception, defined here as the processing of musical sounds from acoustics to neural representations to percepts and distinct from music production, is a central goal in auditory neuroscience."

L504: [magnitude-only auditory spectrogram] specify "(cochleagram)" after spectrogram. Alternatively, use cochleagram throughout the manuscript.

We added a mention to the cochleagram, along with references, on P29. However, we chose to keep using "auditory spectrogram" (and a few times "spectrogram", for convenience) throughout the manuscript, as

it is the name coined in the paper presenting the toolbox we used to compute it (Chi et al., 2005), as well as the name used in further literature (e.g., Pasley et al., 2012; Martin et al., 2017). Moreover, the auditory spectrogram slightly differs from the cochleagram, in that after the cochlear filter bank (yielding the cochleagram), the signal undergoes additional steps such as nonlinear compression, low-pass filtering, derivation, half-wave rectification and temporal integration, and ends up mimicking the output of the auditory midbrain. We agree that the cochleagram and auditory spectrogram are similar, with their most important feature being a cochlea-inspired, logarithmic frequency axis, and recognize the term is also used in the literature (e.g., Norman-Haignere et al., 2022).

P29, L543-547: "This transformation […] outputs a psychoacoustic-, neurophysiologic-based spectrotemporal representation of the song (similar to the cochleagram) (70,71)."

L705: [with i from] with i ranging from

We made this edit in the manuscript, and thank the reviewer for helping us improve the writing (P39, L750).

L710: [most salient coefficient] meaning unclear

We thank the reviewer for catching this equivocal expression, and we completed it in the manuscript by stating the exact mathematical operation and property involved (i.e., highest absolute value, or magnitude).

P39, L755-756: "Then, we determined each component sign by setting as positive the sign of the most salient coefficient (i.e., with the highest absolute value, or magnitude)."
